# Organic anion transporter 2 transcript variant 1 shows broad ligand selectivity when expressed in multiple cell lines

**DOI:** 10.3389/fphar.2015.00216

**Published:** 2015-10-06

**Authors:** Adam G. Hotchkiss, Liam Berrigan, Ryan M. Pelis

**Affiliations:** Department of Pharmacology, Dalhousie UniversityHalifax, NS, Canada

**Keywords:** organic anion transporter 2, transcript variant, multi-selective, drug transporter, solute carrier family, human embryonic kidney cell, Madin-Darby canine kidney cell, Chinese hamster ovary cell

## Abstract

Organic anion transporter 2 (OAT2) is likely important for renal and hepatic drug elimination. Three variants of the OAT2 peptide sequence have been described – OAT2 transcript variant 1 (OAT2-tv1), OAT2 transcript variant 2 (OAT2-tv2), and OAT2 transcript variant 3 (OAT2-tv3). Early studies helping to define the ligand selectivity of OAT2 failed to identify the variant used, and the studies used several heterologous expression systems. In preliminary studies using OAT2-tv1, we failed to observe transport of several previously identified substrates, leading us to speculate that ligand selectivity of OAT2 differs with variant and/or heterologous expression system. The purpose was to further investigate the ligand selectivity of the OAT2 variants expressed in multiple cell types. We cloned OAT2-tv1 and OAT2-tv2, but were unsuccessful at amplifying mRNA for OAT2-tv3 from human kidney. OAT2-tv1 and OAT2-tv2 were individually expressed in human embryonic kidney (HEK), Madin-Darby canine kidney (MDCK), or Chinese hamster ovary (CHO) cells. mRNA for OAT2-tv1 and OAT2-tv2 was demonstrated in each cell type transfected with the respective construct, indicating their expression. OAT2-tv1 trafficked to the plasma membrane of all three cell types, but OAT2-tv2 did not. OAT2-tv1 transported penciclovir in all three cell types, but failed to transport *para*-aminohippurate, succinate, glutarate, estrone-3-sulfate, paclitaxel or dehydroepiandrosterone sulfate – previously identified substrates of OAT2-tv2. Not surprising given its lack of plasma membrane expression, OAT2-tv2 failed to transport any of the organic solutes examined, including penciclovir. Penciclovir transport by OAT2-tv1 was sensitive to large (e.g., cyclosporine A) and small (e.g., allopurinol) organic compounds, as well as organic anions, cations and neutral compounds, highlighting the multiselectivity of OAT2-tv1. The potencies with which indomethacin, furosemide, cyclosporine A and cimetidine inhibited OAT2-tv1 are in good agreement with previous studies using this variant, but inconsistent with studies using OAT2 with an unidentified sequence. This study shows that organic molecules with diverse physicochemical properties interact with OAT2-tv1, making it a likely site of drug interactions. Many previously identified substrates of OAT2 are not transported by OAT2-tv1, suggesting that variant and/or expression system may contribute. Future work should establish the expression pattern and ligand selectivity of OAT2-tv3.

## Introduction

Organic anion transporter 2 (OAT2; SLC22A7) is a member of the SLC22A family, and is closely related to the multi-selective renal organic anion transporters, organic anion transporter 1 (OAT1; SLC22A6) and organic anion transporter 3 (OAT3; SLC22A8). OAT1 and OAT3 each share ∼37% amino acid identity with OAT2. In comparison, OAT1 and OAT3, which have distinct but also overlapping substrate selectivity, share ∼48% amino acid sequence identity to each other. Although OAT2 is present in the kidney, it is predominately expressed in the liver (Sun et al., 2001; Fork et al., 2011). The ligand selectivity and transport mechanism of OAT1 and OAT3 have been well characterized, and much attention has been placed on these transporters (among other select drug transporters) since they are known to influence the pharmacokinetics of numerous clinically relevant drugs, and because they represent potential sites of drug–drug interactions (Giacomini et al., 2010). In contrast, much less is known regarding the ligand selectivity and transport mechanism of OAT2, or its role in pharmacokinetics.

Several variants of the OAT2 peptide sequence have been reported in the literature. Transcript variant 1 (OAT2-tv1; GenBank accession number NP006663) and transcript variant 2 (OAT2-tv2; GenBank accession number NP696961) share 100% amino acid sequence identity, but OAT2-tv1 is two amino acids shorter (Cropp et al., 2008) – OAT2-tv1 and OAT2-tv2 contain 546 and 548 amino acids, respectively. Sun et al. (2001) also identified two transcript variants, and named them hOAT2A and hOAT2B. hOAT2A and hOAT2B were reported to contain 546 and 538 amino acids, respectively, and it was suggested that they are likely alternatively spliced variants, which contain different C-terminal segments (Sun et al., 2001). However, the GenBank accession number AI016020 provided for hOAT2A and hOAT2B (Sun et al., 2001) is linked to a partial Expressed Sequence Tag sequence, and thus, the actual peptide sequences are unknown. Enomoto et al. (2002) reported using an OAT2 construct that contains 539 amino acids. The peptide sequence for this variant is deposited in the National Center for Biotechnology Information (NCBI) database under GenBank accession# AAG43523. For the purpose of this study, we have denoted this construct as OAT2-tv3. Compared to OAT2-tv1, OAT2-tv3 has a phenylalanine instead of leucine at amino acid position 302, and the C-terminal tail is truncated, with 10 out of its last eleven amino acids differing from the corresponding amino acids in OAT2-tv1 – this may in fact be the hOAT2B sequence cloned by Sun et al. (2001). Supplemental Figure S1 shows an amino acid alignment of the three OAT2 sequences identified to date.

Much of our knowledge of the ligand selectivity of OAT2 comes from studies using the cloned transporter expressed in heterologous expression systems. **Table 1** provides a summary of studies that have examined the substrate selectivity of OAT2, showing the OAT2 construct and heterologous expression system used, along with the organic solutes that were identified as substrates. Importantly, many of the studies never cited the OAT2 sequence used, and the studies used several different heterologous expression systems, including S2 cells (immortalized S2 proximal tubule segment from mouse), human embryonic kidney (HEK) cells, Madin-Darby canine kidney (MDCK) cells and *Xenopus laevis* oocytes. More recent studies (2008 and after) examining the ligand selectivity of OAT2 have mostly used OAT2-tv1. These studies demonstrated that OAT2-tv1 is capable of transporting select guanine-containing antivirals (Cheng et al., 2012), creatinine (Shen et al., 2015), orotic acid, and glutamate (Fork et al., 2011), select antineoplastic drugs (Marada et al., 2015) and select guanine nucleotides, including cGMP (Cropp et al., 2008) (**Table 1**).

**Table 1:** Summary of studies that have examined the substrate selectivity of organic anion transporter 2 (OAT2).

Study	Variant	Accession number	Expression system	Substrates
Sun et al., 2001	?	AI016020	HEK293	*para*-aminohippurate, methotrexate, cAMP, α-ketoglutarate, fluorescein
Babu et al., 2002	?		S2 cells	Tetracycline, prostaglandin F2α
Enomoto et al., 2002	tv3	AF210455	S2 cells	Prostaglandin F_2_α
Takeda et al., 2002	?		S2 cells	Prostaglandin F_2_α, zidovudine
Kimura et al., 2002	?		S2 cells	Prostaglandin E_2_
Khamdang et al., 2002	?		S2 cells	Prostaglandin F_2_α, salicylate
Khamdang et al., 2003	?		S2 cells	Prostaglandin F_2_α
Kobayashi et al., 2005a	tv2	AY050498	*Xenopus* oocytes	Estrone-3-sulfate, bumetanide, glutarate, dehydroepiandrosterone sulfate, allopurinol, prostaglandin E_2_, 5-fluorouracil, paclitaxel, ascorbate, *para*-aminohippurate
Kobayashi et al., 2005b	?		*Xenopus* oocytes	Theophylline, erythromycin
Cropp et al., 2008	tv1	NM006672	HEK293	cGMP, 2’-deoxyguanosine and many naturally occurring nucleobases, nucleosides, and nucleotides
Cropp et al., 2008	tv2	NM153320	HEK293	Was not expressed at the plasma membrane
Sato et al., 2010	tv3	AF210455	HEK293	Uric acid
Cheng et al., 2012	tv1	NM006672	HEK293	Penciclovir, acyclovir, ganciclovir
Fork et al., 2011	tv1	NM006672	HEK293	Orotic acid, glutamate, cGMP, 2’-deoxyguanosine, trigonelline
Lepist et al., 2014	?		Madin-Darby canine kidney (MDCK) II	cGMP, creatinine
Shen et al., 2015	tv1	NM006672	HEK293	Penciclovir, cGMP creatinine
Marada et al., 2015	tv1	NM006672	HEK293	cGMP, irinotecan
Babelova et al., 2015	tv1	NM006672	HEK293	cGMP

*The table highlights the OAT2 transcript variant and heterologous expression system used, along with the compounds that were shown to be transported.? indicates that the OAT2 sequence used was not identified in the publication. The GenBank accession numbers shown are for the corresponding nucleotide sequences. The GenBank accession number AI016020 refers to an Expressed Sequence Tag. The GenBank accession numbers for the peptide sequences are as follows – AY050498 (AAL12496), AF210455 (AAG43523), NM006672 (NP006663), and NM153320 (NP696961).*

In preliminary experiments we examined the transport of estrone-3-sulfate and *para*-aminohippurate by OAT2-tv1 expressed in Chinese hamster ovary (CHO) cells, and failed to observe mediated transport, despite previous work indicating that they are OAT2 substrates (**Table 1**). Since these early studies used different heterologous systems and possibly OAT2 variants, we hypothesized that the discrepancy could be due to the OAT2 variant and/or heterologous expression system used. Thus, the purpose of the present study was to examine the ligand selectivity of OAT2 expressed in a variety of mammalian cells, and to compare this data to previously published data. Interestingly, while we were able to clone OAT2-tv1 from human kidney, and obtained the OAT2-tv2 from a commercial source, we were unsuccessful at amplifying mRNA for OAT2-tv3 from human kidney. Thus, we examined ligand selectivity of OAT2-tv1 and OAT2-tv2 in three different mammalian cell lines – CHO, HEK, and MDCK cells.

## Materials and Methods

### Reagents and Chemicals

[^3^H]*para*-aminohippurate (60 Ci/mmol), [^3^H]estrone-3-sulfate (50 Ci/mmol), [^3^H]dehydroepiandrosterone sulfate (63 Ci/mmol), [^14^C]glutarate (55 mCi/mmol), and [^14^C]succinate (54.0 mCi/mmol) were from American Radiochemicals (St. Louis, MO, USA). [^3^H]penciclovir (1.1–18.6 Ci/mmol) and [^3^H]paclitaxel (6.4 Ci/mmol) were from Moravek Biochemicals (Brea, CA, USA). Ham’s F12K medium, Dulbecco’s Modified Eagle’s Medium (DMEM), fetal bovine serum (Certified, US Origin), 1% penicillin-streptomycin solution, zeocin, hygromycin B, Platinum High Fidelity DNA polymerase and 4–12% Tris-glycine gels were from Life Technologies (Burlington, ON, USA). Custom oligonucleotide primers were synthesized by Integrated DNA Technologies (Coralville, IA, USA). The bicinchoninic acid protein assay kit, Sulfo-NHS-SS-biotin reagent, Streptavidin UltraLink resin and the SuperSignal^®^ West Femto chemiluminescent substrate were from Thermo Scientific (Rockford, IL, USA). All other chemicals were obtained from Sigma–Aldrich (St. Louis, MO, USA). Human kidney cortex from a single donor was obtained from the *Eunice Kennedy Shriver* National Institute of Child Health and Human Development (NICHD) Brain and Tissue Bank at the University of Maryland (Baltimore, MD, USA). The use of human kidney tissue was approved by the Research Ethics Board of Dalhousie University. Waymouth buffer (WB) used for transport experiments contained (in mM): 135 NaCl, 28 D-glucose, 5 KCl, 1.2 MgCl_2_, 2.5 CaCl_2_, 0.8 MgSO_4_, and 13 HEPES-NaOH, pH 7.4. Phosphate buffered saline (PBS) containing calcium and magnesium (PBS/CM) used in biotinylation experiments contained (in mM): 137 NaCl, 2.7 KCl, 8 Na_2_HPO_4_, 0.1 CaCl_2_, and 1 MgCl_2_, pH 8.0.

### Cloning of the Human Orthologs of OAT2 Transcript Variants 1 and 2

The open reading frame of the human ortholog of OAT2 transcript variant 1 (OAT2-tv1) containing 546 amino acids was amplified from human kidney cDNA. The open reading frame of the human ortholog of OAT2 transcript variant 2 (OAT2-tv2) containing 548 amino acids was amplified from a construct obtained from Origene (model# SC108270, Rockville, MD, USA) that was contained within the pCMV6-XL4 vector. Platinum High Fidelity DNA Polymerase and sequence-specific oligonucleotide primers were used in the polymerase chain reaction (PCR) reactions. PCR products were purified by gel extraction and the constructs were subcloned into the pcDNA5/FRT/V5-His-TOPO mammalian expression plasmid according to the manufacturer’s protocol. The constructs were designed to contain their native stop codon. Plasmid DNA was prepared using the MO BIO Ultra Clean plasmid preparation kit and DNA sequencing (MCLAB, San Francisco, CA, USA) showed that the OAT2-tv1 and OAT2-tv2 constructs corresponded to GenBank Accession number NM006672 and GenBank Accession number NM153320, respectively, as reported in the NCBI database.

### Cell Culture and Stable Expression of OAT2-tv1 and OAT2-tv2 Cell Lines

Chinese hamster ovary Flp-In cells were grown in medium containing Ham’s F12 Kaighn’s modification medium, 1% penicillin-streptomycin, 10% fetal bovine serum and zeocin (100 μg/ml). HEK Flp-In and MDCK Flp-In cells were grown in DMEM containing 1% penicillin-streptomycin, 10% fetal bovine serum and zeocin (100 μg/ml). CHO, HEK, and MDCK cell lines stably expressing OAT2-tv1 or OAT2-tv2 were generated using electroporation and selection as described previously (Ingraham et al., 2014). The stable OAT2-tv1 and OAT2-tv2 cell lines were grown in the identical medium outlined above, except that the medium contained hygromycin B (200 μg/ml) instead of zeocin. Cells were grown at 37°C in a humidified atmosphere (5% CO_2_/95% air).

### RNA Isolation and Reverse Transcription Polymerase Chain Reaction (RT-PCR)

Total RNA was purified from cells using the RNeasy Mini Kit according to the manufacturer’s instructions. RNA concentration was determined by UV spectrophotometry. RT-PCR was conducted using standard procedures as described previously (Lee et al., 2015). The oligonucleotide primer sequences used for amplifying the human ortholog of OAT2-tv1 and OAT2-tv2 were: 5′-CATTGCAACTGAGTCCCAGTG-3′ (sense) and 5′-CAGGAGGAAGTGCAGTGGTA -3′ (antisense).

### Cell Surface Biotinylation and Western Blotting

Cell surface biotinylation was conducted with cells grown in one well of a 12-well plate. Cell surface biotinylation was performed using Sulfo-NHS-SS-biotin (0.5 mg/ml) using procedures identical to that described previously (Astorga et al., 2011). The biotinylated proteins were separated on 4–12% tris-glycine gels, and transferred to polyvinylidene difluoride membranes. The antibodies used were a polyclonal rabbit anti-human OAT2 antibody (0.5 μg/ml) (Cosmo Bio Ltd., Carlsbad, CA, USA) and a goat anti-rabbit HRP-conjugated secondary antibody (4 μg/ml). The SuperSignal West Femto chemiluminescent (Thermo Scientific) substrate was used for immunoreactivity detection.

### Transport Studies Examining Cellular Uptake

All experiments examining cellular radiolabeled compound uptake were conducted using cells grown to confluence in 24-well flat bottom plates. The transport solution consisted of WB (room temperature) containing radiolabeled compound and the uptake period for all experiments was at an initial rate time point of 5 min. [^3^H]Penciclovir served as the probe OAT2 substrate. We also examined the uptake of [^3^H]*para*-aminohippurate, [^3^H]estrone-3-sulfate, [^14^C]glutarate, [^14^C]succinate, [^3^H]paclitaxel, and [^3^H]dehydroepiandrosterone sulfate by OAT2 since published studies indicate that they are substrates of the transport protein (Sun et al., 2001; Kobayashi et al., 2005a). To examine the potential for compounds to inhibit OAT2, the transport solution contained [^3^H]penciclovir and either a fixed concentration of test compound (1 mM), or increasing concentrations of test compound to determine potency of inhibition (inhibitor concentration 50, IC_50_). For all cellular uptake experiments, the medium was aspirated and the cells were rinsed once with WB (0.3 ml). Following the uptake period, cells were washed three times with ice-cold WB (0.5 ml each wash). After aspirating the final wash the cells were lysed in 0.5 N NaOH/1% SDS (0.4 ml) for ∼30 min on an orbital shaker. 1 N HCl (0.2 ml) was then added to neutralize the NaOH, and the cell lysates (0.5 ml) were transferred to scintillation vials containing liquid scintillation cocktail (CytoScint ES, MP Biomedicals). Cellular radioactivity content was determined with a Beckman LS6500 liquid scintillation counter. Protein content was determined using the bicinchoninic acid method. See the Figure and Table Legends for additional details describing the experimental conditions used.

### Data Analysis

Transport data are reported as the mean ± standard error of the mean of at least three independent experiments using cells of a different passage number. A two-tailed unpaired Student’s *t-*test was used for comparisons of means between two groups. Significance for all analyses was assigned at *P* < 0.05. All graphing, non-linear regression analyses and statistical analyses were performed with GraphPad Prism (version 6.03).

## Results

**FIGURE 1:**
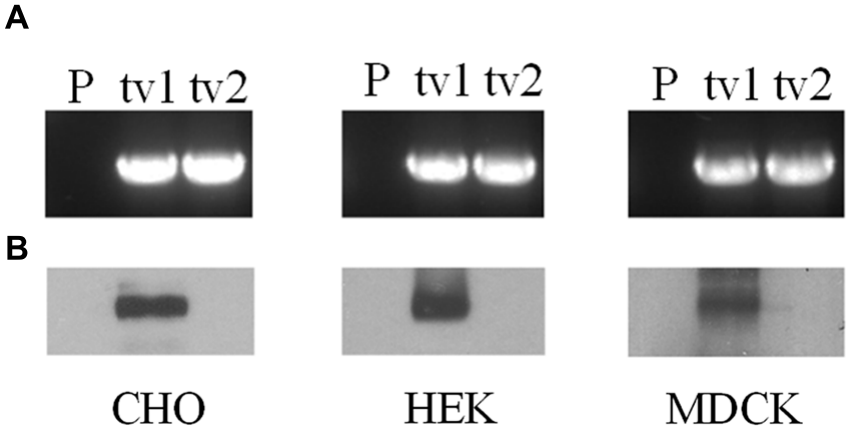
**mRNA expression **(A)** and cell surface protein expression **(B)** of Organic anion transporter 2 (OAT2)-tv1 (tv1) or OAT2-tv2 (tv2) in Chinese hamster ovary (CHO), human embryonic kidney (HEK), or Madin-Darby canine kidney (MDCK) cells stably transfected with the respective construct, or in parental cells (P).** mRNA expression was determined by reverse transcription polymerase chain reaction (RT-PCR) using oligonucleotide primers against OAT2. Amplified products were separated on 1% agarose gels and stained with ethidium bromide. Sulfo-NHS-SS-biotin was used for cell surface biotinylation.

Organic anion transporter 2-tv1 and OAT2-tv2 were individually transfected into CHO, HEK, and MDCK cells, and placed under selection pressure in order to establish stable cell lines. RT-PCR analyses revealed expression of both OAT2-tv1 and OAT2-tv2 at the mRNA level in all three cell types, indicating successful stable expression of both constructs (**Figure 1A**). However, cell surface biotinylation experiments showed that only OAT2-tv1 was present at the cell surface (**Figure 1B**), pointing to a potential trafficking defect associated with OAT2-tv2.

**FIGURE 2:**
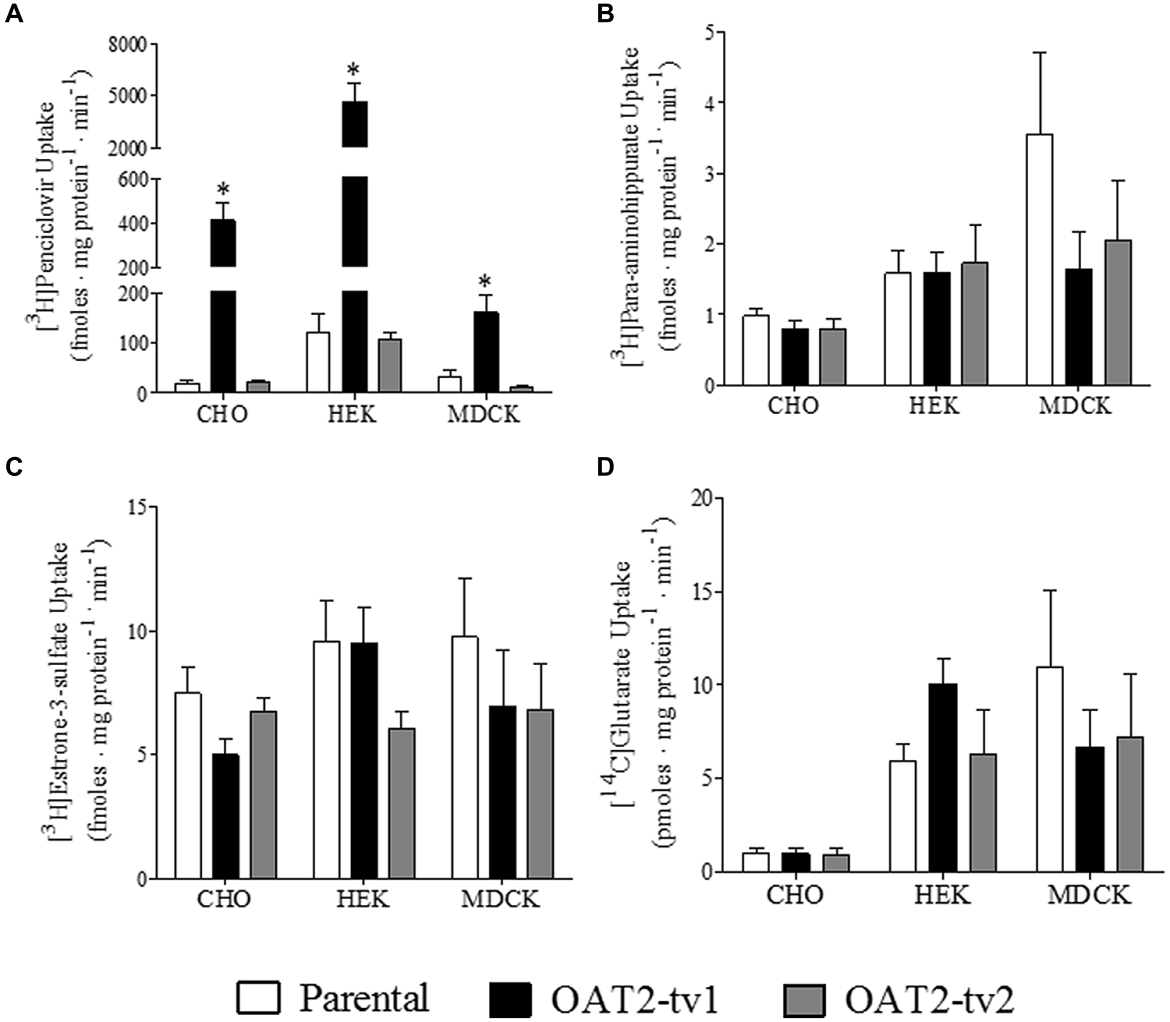
**Cellular accumulation of [^3^H]penciclovir **(A)**, [^3^H]*para*-aminohippurate **(B)**, [^3^H]estrone-3-sulfate **(C)**, or [^14^C]glutarate **(D)** by parental CHO, HEK, or MDCK cells, or CHO, HEK, or MDCK cells stably expressing either OAT2-tv1 or OAT2-tv2.** The concentration of [^3^H]penciclovir in the transport solution was 0.1 μM for CHO and MDCK cell experiments, and 0.5 μM for HEK cell experiments. The concentrations of [^3^H]*para*-aminohippurate, [^3^H]estrone-3-sulfate and [^14^C]glutarate were 8 nM, 10 nM, and 18 μM, respectively. Uptake was conducted for 5 min at room temperature. Values are mean ± standard error of the mean of four experiments. *Significantly different from parental cells, *P* < 0.05, two-tailed unpaired student’s *t*-test.

The uptake of several putative OAT2 substrates was tested in the three different cell types (CHO, HEK, and MDCK) expressing the human orthologs of either OAT2-tv1 or OAT2-tv2 (**Figure 2**). Compared to parental cells, the uptake of penciclovir was 21-fold, 39-fold, and 5-fold significantly higher in CHO-OAT2-tv1, HEK-OAT2-tv1, and MDCK-OAT2-tv1 cells, respectively (**Figure 2A**). Consistent with its lack of cell surface expression, the uptake of penciclovir into CHO-OAT2-tv2, HEK-OAT2-tv2, and MDCK-OAT2-tv2 cells was not different from uptake into the respective parental cells (**Figure 2A**). The uptake of *para*-aminohippurate (**Figure 2B**), estrone-3-sulfate (**Figure 2C**), or glutarate (**Figure 2D**) into CHO, HEK, and MDCK cells expressing either OAT2-tv1 or OAT2-tv2 was not different than uptake into the respective parental cells. Additionally, when expressed in CHO cells, OAT2-tv1 was unable to show mediated transport of two other putative OAT2 substrates, dehydroepiandrosterone sulfate and paclitaxel. The cellular accumulation of [^3^H]dehydroepiandrosterone sulfate (12 nM) into CHO parental vs. CHO-OAT2-tv1 cells was 10.3 ± 1.1 fmoles mg protein^-1^ ⋅ min^-1^ vs. 11.0 ± 0.54 fmoles ⋅ mg protein^-1^ ⋅ min^-1^, respectively (*n* = 3). The cellular accumulation of [^3^H]paclitaxel (22 nM) into CHO parental vs. CHO-OAT2-tv1 cells was 89 ± 3.8 fmoles ⋅ mg protein^-1^ ⋅ min^-1^ vs. 90 ± 5.1 fmoles ⋅ mg protein^-1^ ⋅ min^-1^ (*n* = 3). Given the similarity in substrate selectivity of OAT2-tv1 regardless of cell type, and the lack of functional expression of OAT2-tv2, all subsequent experiments were done with the CHO-OAT2-tv1 cells.

**FIGURE 3:**
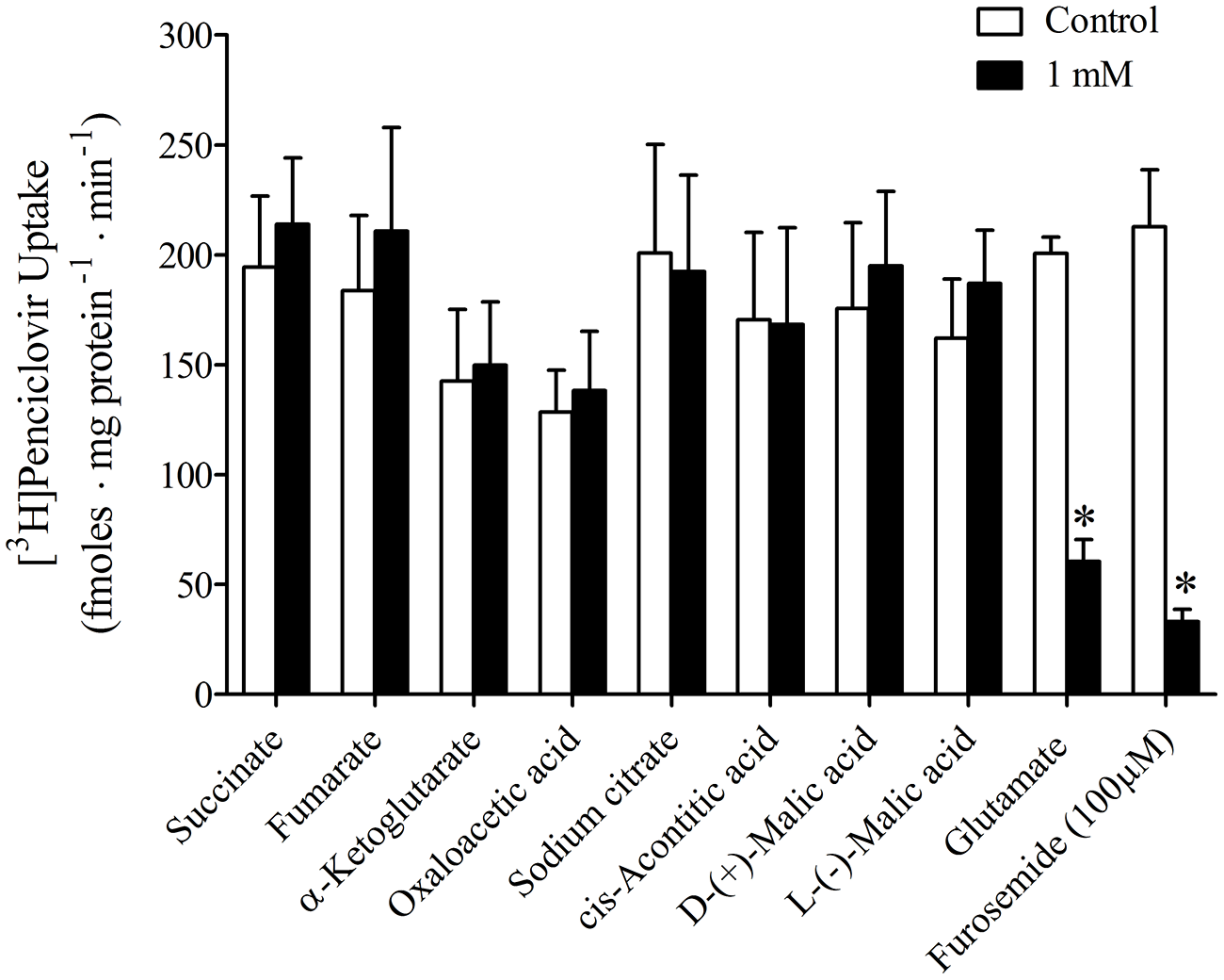
***Cis*-inhibition of [^3^H]penciclovir (∼30 nM) uptake into CHO-OAT2-tv1 cells by Kreb’s cycle intermediates.** The concentration of each Kreb’s cycle intermediate was 1 mM. Uptake was conducted for 5 min. Glutamate (1 mM) or furosemide (200 μM) were used as positive control inhibitors. Values are the mean ± standard error of the mean from 3 to 4 experiments. *Significantly different from control (open bar), *P* < 0.05, two-tailed unpaired Student’s *t-*test.

**FIGURE 4:**
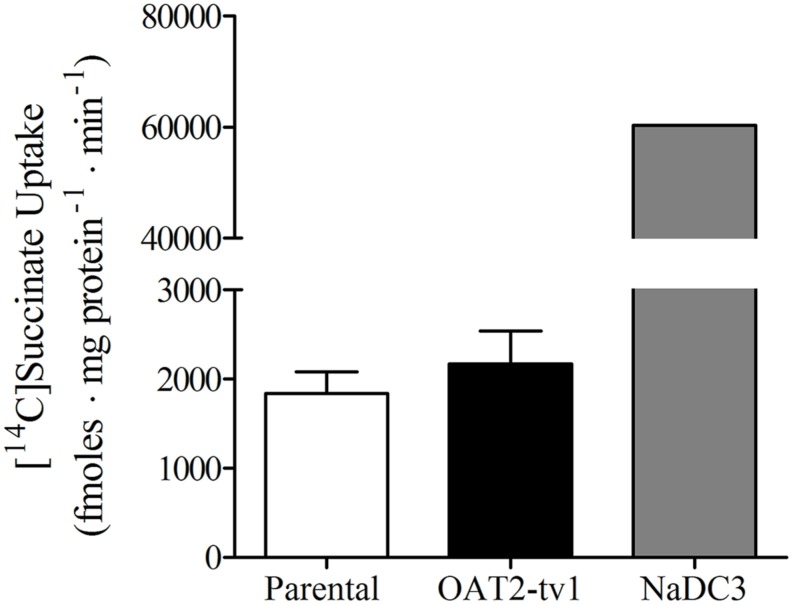
**Cellular accumulation of [^14^C]succinate (18.5 μM) by CHO parental cells, CHO-OAT2-tv1 cells or CHO cells stably expressing the Na-dicarboxylate cotransporter 3 (NaDC3).** Uptake was conducted for 5 min. Values are the mean ± standard error of the mean of four experiments for CHO parental and CHO-OAT2-tv1 cells. A single uptake experiment was performed with CHO-NaDC3 cells. There was no significant difference in [^14^C]succinate uptake by CHO-OAT2-tv1 cells compared to parental CHO cells (*P* > 0.05, two-tailed unpaired student’s *t*-test.

It was previously reported that succinate and fumarate are capable of trans*-*stimulating OAT2-mediated substrate uptake (Kobayashi et al., 2005a), suggesting that OAT2 may be an organic anion/dicarboxylate exchanger. Given these previous results we hypothesized that if dicarboxylates interact as substrates of OAT2 that they may also inhibit the transport protein. Thus, we examined the ability of a variety of Kreb’s cycle intermediates (succinate, fumarate, α-ketoglutarate, oxaloacetate, citrate, *cis*-aconitate, and D-malate) to *cis*-inhibit penciclovir uptake into CHO-OAT2-tv1 cells. Even at a high concentration (1 mM), none of the Kreb’s cycle intermediates tested, including succinate and fumarate, *cis*-inhibited penciclovir uptake (**Figure 3**). Conversely, the OAT2 inhibitors glutamate (1 mM) and furosemide (200 μM) reduced penciclovir uptake by 70–85% compared to control, confirming that OAT2-tv1 was functional (**Figure 3**). We also examined the uptake of [^14^C]-succinate by CHO-OAT2-tv1 cells to determine if it is in fact a substrate. The uptake of [^14^C]succinate by CHO-OAT2-tv1 cells was not different than uptake into the parental CHO cells (**Figure 4**). In a single experiment (*n* = 1), the uptake of [^14^C]succinate by CHO cells stably expressing the Na-dicarboxylate cotransporter 3 (NaDC3) was ∼30-fold higher than control.

Given the results that previously identified substrates (*para*-aminohippurate, estrone-3-sulfate, glutarate, dehydroepiandro sterone sulfate, paclitaxel, and succinate) were not transported by OAT2-tv1 in the present study led us to examine the inhibitory potential of a variety of drugs (organic anions, cations, and neutral molecules) against OAT2-tv1. A total of seventeen compounds with diverse physicochemical properties were tested for their potency to inhibit penciclovir uptake by CHO-OAT2-tv1 cells (**Table 2**). The majority of organic anions tested were effective inhibitors, reducing penciclovir uptake by ≥85% of the control level. These compounds inhibited with varying degrees of potency ranging from 3.7 μM (indomethacin) to 210 μM (*para*-aminohippurate). Methotrexate (76%), probenecid (64%), and cidofovir (53%) were also capable of reducing OAT2-tv1-mediated penciclovir uptake, but the effect was not dose-dependent, so IC_50_ values could not be determined. The organic cations and neutral compounds also showed varying abilities to inhibit OAT2-tv1. Notably, cimetidine and allopurinol each inhibited 95% of transport activity, with IC_50_ values of 57 and 70 μM, respectively.

**Table 2:** Potency of OAT2-tv1 inhibition by organic molecules with diverse physicochemical properties.

Test compound	Maximum concentration (μM)	% Inhibition	IC_50_ (μM)	MW
**Organic anions**				
Indomethacin	1000	99	3.7 ± 0.5	357.8
Repaglinide	500	99	30.0 ± 3.6	452.6
*para-*aminohippurate	1000	99	210.3 ± 77	194.2
Telmisartan	200	96	17.1 ± 1.3	514.6
Prostaglandin E2	500	96	26.8 ± 2.4	352.5
Estrone Sulfate	1000	95	36.1 ± 3.9	372.4
Furosemide	200	90	10.8 ± 1.5	330.7
Valsartan	1000	86	126.9 ± 29	435.5
Methotrexate	1000	76	ND	454.4
Probenecid	1000	64	ND	285.4
Cidofovir	1000	53	ND	279.2
**Organic cations**				
Cimetidine	1000	95	57.2 ± 14	252.3
Erythromycin	1000	65	12.0 ± 5.4	733.9
Cyclosporin A	62.5	55	8.6 ± 4.9	1202.6
Tetrapentylammonium	1000	49	278.8 ± 142	378.5
Tetraethylammonium	1000	42	54.3 ± 33	210.2
**Neutral compounds**				
Allopurinol	1000	95	70.1 ± 34	136.1
Penciclovir	1500	85	78.4 ± 8.2	253.3

*[^*3*^H] Penciclovir (∼30 nM) was the substrate. Uptake was conducted for 5 min at room temperature in the presence of increasing concentrations of test compound. The maximum concentration of test compound used is indicated. The potency of inhibition (IC_*50*_ determination) was determined by non-linear regression analysis. IC_*50*_ values are the mean ± standard error of the mean of 3–4 experiments. The % inhibition corresponds to the percent reduction in transport, compared to parental cells, that was achieved by the maximum drug concentration tested. ND, the IC_*50*_ could not be determined by non-linear regression analysis. MW, test compound molecular weight (grams/mol).*

## Discussion

In the present study we cloned and stably expressed OAT2-tv1 and OAT2-tv2 in three different mammalian cell lines in order to examine their ligand selectivity. Unexpectedly, several attempts to amplify OAT2-tv3 mRNA from human kidney were unsuccessful. OAT2-tv3 was cloned by Enomoto et al. (2002), and in their manuscript they reference GenBank accession# AF210455 as the corresponding nucleotide sequence. According to NCBI, this OAT2 construct was originally cloned from human kidney with the authorship listed as: Reid G, Bahn A, Ebbinghaus C, Wolff NA, and Burckhardt G (deposited in 2001). Also of interest, there are several studies that have examined the ligand selectivity of OAT2, where the sequence used was not cited [(Sun et al., 2001; Babu et al., 2002; Khamdang et al., 2002, 2003; Kimura et al., 2002; Takeda et al., 2002; Kobayashi et al., 2005b; Lepist et al., 2014); **Table 1**]. Like the Enomoto et al. (2002) study that described the cloning of OAT2-tv3, most of these other studies had the same senior author, suggesting that OAT2-tv3 may have been used in these other studies as well. However, since the exact sequence used was not cited in these manuscripts, we cannot be certain that this is the case.

OAT2-tv3 shares 97% sequence identity with OAT2-tv1 and OAT2-tv2. The OAT2-tv3 amino acid sequence differs from OAT2-tv1 and OAT2-tv2 primarily in its C-terminal end (Supplemental Figure S1). Amino acid alignment of OAT2 from human (OAT2-tv1 and OAT2-tv3), mouse, rat, rabbit, cattle, pig, horse, opossum, and chicken indicates that the C-terminal end varies across species as well (Supplemental Figure S2). Interestingly, there are a greater number of conserved amino acids at homologous positions in the C-terminal of OAT2 from other species (except mouse) and OAT2-tv1, than with OAT2-tv3 (Supplemental Figure S2). A BLAST search of the human genome in NCBI using the OAT2-tv3 cDNA sequence as the query results in OAT2-tv1 and OAT2-tv2 as hits, but not OAT2-tv3. The OAT2-tv3 sequence was also not found in the NHLBI Exome Sequencing Project Exome Variant Server database (http://evs.gs.washington.edu/EVS/). Future studies should be directed at determining if OAT2-tv3 actually exists, and if so, its tissue, cellular and subcellular distribution, as well as how its sequence variation affects ligand selectivity.

When expressed in either CHO, MDCK, or HEK cells, OAT2-tv2 was non-functional. While OAT2-tv2 expression at the mRNA level in each of the cell lines was detected, cell surface biotinylation experiments indicated that the protein was not present at the plasma membrane, unlike OAT2-tv1, which showed clear plasma membrane expression. OAT2-tv2 has an additional serine and glutamine at amino acid positions 132 and 133, respectively. Homology modeling was previously used to predict the tertiary structure of OAT1 (Perry et al., 2006), which is related to OAT2. Supplemental Figure S3 shows an amino acid alignment between the first ∼200 amino acids of OAT1, OAT2-tv1, and OAT2-tv2. The figure highlights the position of transmembrane helices 1 and 2, as well as the long extracellular loop in the OAT1 model. Assuming similar topologies between OAT1 and OAT2, the additional two amino acids in OAT2-tv2 are expected to occur in the long extracellular loop between transmembrane helices 1 and 2 (Supplemental Figure S3) – these two amino acids were also predicted to be in the putative long extracellular loop of OAT2 using HMMTOP 2.0. Several studies have shown that modification of amino acids in the long extracellular loop of OAT1 (as well as other related SLC drug transporters) has a profound influence on its plasma membrane expression (Tanaka et al., 2004a,b), and given these results, the insertion of two additional amino acids in the long loop of OAT2 may affect its protein folding and trafficking. Consistent with this hypothesis, Cropp et al. (2008) showed by immunocytochemistry that OAT2-tv2 protein in HEK cells is retained in an intracellular compartment. Alternatively, the OAT2-tv2 variant may have an increased rate of protein degradation (Cropp et al., 2008). While OAT2-tv2 shows no apparent expression at the plasma membrane of any mammalian cell tested to date, it was clearly expressed and functional in *X. laevis* oocytes (Kobayashi et al., 2005a) (**Table 1**). This raises the question of the most appropriate heterologous system for studying mammalian transport proteins, and what the functional role, if any, OAT2-tv2 has in human tissues. Cropp et al. (2008) showed that mRNA for OAT2-tv2 is expressed at similar levels to OAT2-tv1 in a variety of tissues. Perhaps OAT2-tv2 has a role in transporting its substrates across intracellular membranous compartments.

Several early studies that have helped define the substrate selectivity of human OAT2 were done using a construct with an undefined sequence or OAT2-tv2 expressed in *Xenopus* oocytes (**Table 1**), which is non-functional in all mammalian cells tested to date. In particular, early studies have suggested that *para*-aminohippurate, estrone-3-sulfate, glutarate, dehydroepiandrosterone sulfate, paclitaxel, among others, are human OAT2 substrates (**Table 1**). Importantly, according to nearly all reviews on organic anion transport, the aforementioned compounds are listed as substrates of human OAT2 [e.g., (Vanwert et al., 2010)]. Yet, we showed that OAT2-tv1 does not transport these organic anions in the cell types tested, but did transport penciclovir in all three cell types. Together, these data indicate that the substrate selectivity of OAT2-tv1 is similar in CHO, HEK, and MDCK cells. Cropp et al. (2008) also failed to observe appreciable uptake of *para*-aminohippurate by HEK-OAT2-tv1 cells. Also consistent with our results, Sun et al. (2001) did not observe transport of dehydroepiandrosterone sulfate by OAT2 – although it is unclear what variant was used in their study. Despite not observing transport of *para*-aminohippurate, estrone-3-sulfate, glutarate, dehydroepiandrosterone sulfate, or paclitaxel, we did show robust transport of penciclovir by OAT2-tv1, which has been demonstrated previously (Cheng et al., 2012; Shen et al., 2015). When expressed in S2 cells, OAT2 (sequence not given) failed to transport acyclovir or ganciclovir (Takeda et al., 2002), yet, these antivirals, along with penciclovir, are clearly substrates of OAT2-tv1 (Cheng et al., 2012). Interestingly, most recent studies examining ligand selectivity of OAT2 have used transcript variant 1, as opposed to the other variants (**Table 1**).

Information regarding the transport mechanism of OAT2 is limited to only a few studies that used either human, mouse or rat orthologs of OAT2. Transport by all three orthologs is Na-independent (Sekine et al., 1998; Kobayashi et al., 2002, 2005a). Fork et al. (2011) showed that OAT2-tv1 in HEK cells can mediate orotic acid/orotic acid and orotic acid/glutamate exchange. Uptake of estrone-3-sulfate by OAT2-tv2 in *Xenopus* oocytes was modestly stimulated by pre-loading the oocytes with 5 mM fumarate (1.7-fold stimulation) or succinate (2.1-fold), suggesting an anion exchange mechanism involving either of these Kreb’s cycle intermediates (Kobayashi et al., 2005a). In contrast, Sato et al. (2010) failed to observe stimulation of urate uptake by OAT2-tv3 in HEK cells pre-loaded with succinate (100 mM). Given these discrepancies with succinate, we tested the *cis*-inhibitory effect of a variety of Kreb’s cycle intermediates (including fumarate and succinate) with OAT2-tv1. However, none of the Kreb’s cycle intermediates tested (at 1 mM) inhibited transport of penciclovir by OAT2-tv1. Similar to our results, Babelova et al. (2015) did not observe *cis*-inhibition of OAT2-tv1-mediated cGMP transport by citrate or *cis*-aconitate. We further showed that, indeed, [^14^C]succinate is not transported by OAT2-tv1 in CHO cells. Our data, along with those of Sato et al. (2010), suggest that at least succinate is not translocated by OAT2-tv1. This raises the question – what is the driving force for OAT2-mediated substrate uptake into cells? Glutamate is an abundant intracellular amino acid (2–20 mM; Newsholme et al., 2003), and Fork et al. (2011) showed that the outwardly directed glutamate gradient can stimulate OAT2-tv1-mediated substrate uptake via an exchange mechanism. Thus, as previously suggested (Fork et al., 2011), glutamate is a good candidate for a physiological trans-substrate for OAT2-tv1-mediated translocation.

Given that several previously identified substrates were not transported by OAT2-tv1 in the present study, we also examined the inhibitory potential of a variety of organic compounds against OAT2-tv1. To our knowledge, many of the compounds tested have not been previously examined for their inhibition potency against OAT2, including repaglinide, *para*-aminohippurate, telmisartan, estrone-3-sulfate, valsartan, methotrexate, cidofovir, tetrapentylammonium, tetraethylammonium, and allopurinol. The IC_50_ values that we obtained for inhibition of OAT2-tv1 by indomethacin (3.7 μM), furosemide (10.8 μM), cyclosporine A (8.6 μM), and cimetidine (57.2 μM) are in good agreement with other studies examining the potency with which these compounds inhibit OAT2-tv1 – indomethacin [2.1 μM; (Shen et al., 2015)], furosemide [10.9 μM; (Babelova et al., 2015)], cyclosporine A [11.1 μM; (Shen et al., 2015)], and cimetidine [62.3; (Shen et al., 2015)]. In contrast, there is an inconsistency between our data and studies examining the inhibitory potency of several of these compounds against OAT2 with an unidentified sequence – indomethacin [64.1 μM; (Khamdang et al., 2002)], furosemide [603 μM; (Hasannejad et al., 2004)] and cimetidine [no inhibition; (Khamdang et al., 2004)]. Also at odds is the observation that allopurinol is a substrate (Cropp et al., 2008) and inhibitor (present study) of OAT2-tv1, but did not inhibit uric acid transport by OAT2-tv3 in HEK293 cells (Sato et al., 2010). Our data also showed that transport of penciclovir by OAT2-tv1 is sensitive to large (e.g., cyclosporine A) and small (e.g., allopurinol) organic compounds, and organic anions, cations as well as neutral compounds, highlighting the multiselectivity of the OAT2-tv1 ligand binding surface.

In summary, data from the literature on ligand selectivity of OAT2 is complicated by the fact that there are apparently three transcript variants, and many of the studies failed to identify the transcript variant that was used. Previously identified substrates of OAT2-tv2 expressed in *Xenopus* oocytes, including *para*-aminohippurate, succinate, glutarate, estrone-3-sulfate, paclitaxel, or dehydroepiandrosterone sulfate, are not transported by OAT2-tv1. For the limited number of compounds tested, the substrate selectivity of OAT2-tv1 was qualitatively similar regardless of the cell type in which it was expressed. Interestingly, OAT2-tv2 did not express at the plasma membrane of any of the three mammalian cell types examined. There was also a discrepancy between potency of several compounds to inhibit OAT2-tv1 in the present study, and previously published data using OAT2 with an unidentified sequence. OAT2-tv1 was inhibited by compounds with diverse physicochemical properties, making it a potential site of drug–drug interactions. Future studies using OAT2 for ligand interaction studies should identify the transcript variant being used. Additional studies are required to determine the expression pattern and functional activity of OAT2-tv3.

## Author Contributions

Participated in research design: RP and AH.

Conducted experiments: AH and LB.

Performed data analysis: AH and LB.

Wrote or contributed to the writing of the manuscript: RP and AH.

## Conflict of Interest Statement

The authors declare that the research was conducted in the absence of any commercial or financial relationships that could be construed as a potential conflict of interest.

## References

[B1] Astorga B. Wunz T. M. Morales M. Wright S. H. Pelis R. M. (2011). Differences in the substrate binding regions of renal organic anion transporters 1 (OAT1) and 3 (OAT3). *Am. J. Physiol. Renal Physiol.* 301 F378–F386. 10.1152/ajprenal.00735.2010 21543413 PMC3154592

[B2] Babelova A. Burckhardt B. C. Wegner W. Burckhardt G. Henjakovic M. (2015). Sex-differences in renal expression of selected transporters and transcription factors in lean and obese Zucker spontaneously hypertensive fatty rats. *J. Diabetes Res.* 2015 483238 10.1155/2015/483238 PMC4325971 25710042

[B3] Babu E. Takeda M. Narikawa S. Kobayashi Y. Yamamoto T. Cha S. H. (2002). Human organic anion transporters mediate the transport of tetracycline. *Jpn. J. Pharmacol.* 88 69–76. 10.1254/jjp.88.69 11855680

[B4] Cheng Y. Vapurcuyan A. Shahidullah M. Aleksunes L. M. Pelis R. M. (2012). Expression of organic anion transporter 2 in the human kidney and its potential role in the tubular secretion of guanine-containing antiviral drugs. *Drug Metab. Dispos.* 40 617–624. 10.1124/dmd.111.042036 22190696

[B5] Cropp C. D. Komori T. Shima J. E. Urban T. J. Yee S. W. More S. S. (2008). Organic anion transporter 2 (SLC22A7) is a facilitative transporter of cGMP. *Mol. Pharmacol.* 73 1151–1158. 10.1124/mol.107.043117 18216183 PMC2698938

[B6] Enomoto A. Takeda M. Shimoda M. Narikawa S. Kobayashi Y. Kobayashi Y. (2002). Interaction of human organic anion transporters 2 and 4 with organic anion transport inhibitors. *J. Pharmacol. Exp. Ther.* 301 797–802. 10.1124/jpet.301.3.797 12023506

[B7] Fork C. Bauer T. Golz S. Geerts A. Weiland J. Del T. D. (2011). OAT2 catalyses eﬄux of glutamate and uptake of orotic acid. *Biochem. J.* 436 305–312. 10.1042/BJ20101904 21446918

[B8] Giacomini K. M. Huang S. M. Tweedie D. J. Benet L. Z. Brouwer K. L. Chu X. (2010). Membrane transporters in drug development. *Nat. Rev. Drug Discov.* 9 215–236. 10.1038/nrd3028 20190787 PMC3326076

[B9] Hasannejad H. Takeda M. Taki K. Jung S. H. Babu E. Jutabha P. (2004). Interactions of human organic anion transporters with diuretics. *J. Pharmacol. Exp. Ther.* 308 1021–1029. 10.1124/jpet.103.059139 14610216

[B10] Ingraham L. Li M. Renfro J. L. Parker S. Vapurcuyan A. Hanna I. (2014). A plasma concentration of alpha-ketoglutarate influences the kinetic interaction of ligands with organic anion transporter 1. *Mol. Pharmacol.* 86 86–95. 10.1124/mol.114.091777 24770989

[B11] Khamdang S. Takeda M. Babu E. Noshiro R. Onozato M. L. Tojo A. (2003). Interaction of human and rat organic anion transporter 2 with various cephalosporin antibiotics. *Eur. J. Pharmacol.* 465 1–7. 10.1016/S0014-2999(03)01381-5 12650826

[B12] Khamdang S. Takeda M. Noshiro R. Narikawa S. Enomoto A. Anzai N. (2002). Interactions of human organic anion transporters and human organic cation transporters with nonsteroidal anti-inflammatory drugs. *J. Pharmacol. Exp. Ther.* 303 534–539. 10.1124/jpet.102.037580 12388633

[B13] Khamdang S. Takeda M. Shimoda M. Noshiro R. Narikawa S. Huang X. L. (2004). Interactions of human- and rat-organic anion transporters with pravastatin and cimetidine. *J. Pharmacol. Sci.* 94 197–202. 10.1254/jphs.94.197 14978359

[B14] Kimura H. Takeda M. Narikawa S. Enomoto A. Ichida K. Endou H. (2002). Human organic anion transporters and human organic cation transporters mediate renal transport of prostaglandins. *J. Pharmacol. Exp. Ther.* 301 293–298. 10.1124/jpet.301.1.293 11907186

[B15] Kobayashi Y. Ohshiro N. Sakai R. Ohbayashi M. Kohyama N. Yamamoto T. (2005a). Transport mechanism and substrate specificity of human organic anion transporter 2 (hOat2 [SLC22A7]). *J. Pharm. Pharmacol.* 57 573–578. 10.1211/0022357055966 15901346

[B16] Kobayashi Y. Sakai R. Ohshiro N. Ohbayashi M. Kohyama N. Yamamoto T. (2005b). Possible involvement of organic anion transporter 2 on the interaction of theophylline with erythromycin in the human liver. *Drug Metab. Dispos.* 33 619–622. 10.1124/dmd.104.003301 15708966

[B17] Kobayashi Y. Ohshiro N. Shibusawa A. Sasaki T. Tokuyama S. Sekine T. (2002). Isolation, characterization and differential gene expression of multispecific organic anion transporter 2 in mice. *Mol. Pharmacol.* 62 7–14. 10.1124/mol.62.1.7 12065749

[B18] Lee J. Shahidullah M. Hotchkiss A. Coca-Prados M. Delamere N. A. Pelis R. M. (2015). A renal-like organic anion transport system in the ciliary epithelium of the bovine and human eye. *Mol. Pharmacol.* 87 697–705. 10.1124/mol.114.096578 25661037 PMC6067639

[B19] Lepist E. I. Zhang X. Hao J. Huang J. Kosaka A. Birkus G. (2014). Contribution of the organic anion transporter OAT2 to the renal active tubular secretion of creatinine and mechanism for serum creatinine elevations caused by cobicistat. *Kidney Int.* 86 350–357. 10.1038/ki.2014.66 24646860 PMC4120670

[B20] Marada V. V. Florl S. Kuhne A. Muller J. Burckhardt G. Hagos Y. (2015). Interaction of human organic anion transporter 2 (OAT2) and sodium taurocholate cotransporting polypeptide (NTCP) with antineoplastic drugs. *Pharmacol. Res.* 91 78–87. 10.1016/j.phrs.2014.11.002 25481222

[B21] Newsholme P. Procopio J. Lima M. M. Pithon-Curi T. C. Curi R. (2003). Glutamine and glutamate–their central role in cell metabolism and function. *Cell Biochem. Funct.* 21 1–9. 10.1002/cbf.1003 12579515

[B22] Perry J. L. Dembla-Rajpal N. Hall L. A. Pritchard J. B. (2006). A three-dimensional model of human organic anion transporter 1: aromatic amino acids required for substrate transport. *J. Biol. Chem.* 281 38071–38079. 10.1074/jbc.M608834200 17038320 PMC1847411

[B23] Sato M. Mamada H. Anzai N. Shirasaka Y. Nakanishi T. Tamai I. (2010). Renal secretion of uric acid by organic anion transporter 2 (OAT2/SLC22A7) in human. *Biol. Pharm. Bull.* 33 498–503. 10.1248/bpb.33.498 20190416

[B24] Sekine T. Cha S. H. Tsuda M. Apiwattanakul N. Nakajima N. Kanai Y. (1998). Identification of multispecific organic anion transporter 2 expressed predominantly in the liver. *FEBS Lett.* 429 179–182. 10.1016/S0014-5793(98)00585-7 9650585

[B25] Shen H. Liu T. Morse B. L. Zhao Y. Zhang Y. Qiu X. (2015). Characterization of organic anion transporter 2 (SLC22A7): a highly efficient transporter for creatinine and species-dependent renal tubular expression. *Drug Metab. Dispos.* 43 984–993. 10.1124/dmd.114.062364 25904762

[B26] Sun W. Wu R. R. van Poelje P. D. Erion M. D. (2001). Isolation of a family of organic anion transporters from human liver and kidney. *Biochem. Biophys. Res. Commun.* 283 417–422. 10.1006/bbrc.2001.4774 11327718

[B27] Takeda M. Khamdang S. Narikawa S. Kimura H. Kobayashi Y. Yamamoto T. (2002). Human organic anion transporters and human organic cation transporters mediate renal antiviral transport. *J. Pharmacol. Exp. Ther.* 300 918–924. 10.1124/jpet.300.3.918 11861798

[B28] Tanaka K. Xu W. Zhou F. You G. (2004a). Role of glycosylation in the organic anion transporter OAT1. *J. Biol. Chem.* 279 14961–14966. 10.1074/jbc.M400197200 14749323

[B29] Tanaka K. Zhou F. Kuze K. You G. (2004b). Cysteine residues in the organic anion transporter mOAT1. *Biochem. J.* 380 283–287. 10.1042/bj20031724 14979872 PMC1224163

[B30] Vanwert A. L. Gionfriddo M. R. Sweet D. H. (2010). Organic anion transporters: discovery, pharmacology, regulation and roles in pathophysiology. *Biopharm. Drug Dispos.* 31 1–71. 10.1002/bdd.693 19953504

