# Supplementary material for: Organic anion transporter 2 transcript variant 1 shows broad ligand selectivity when expressed in multiple cell lines
Source: Front Pharmacol. 2015 Oct 6;6:216. doi: 10.3389/fphar.2015.00216 (PMC4594013; doi:10.3389/fphar.2015.00216)
Supplement: Supplementary file 1 [file Image_1.PDF]

Supplemental Figure 1

```
OAT2-tv1        MGFEELLEQVGGFGPFQLRNVALLALPRVLLPLHFLLPIFLAAVPAHRCALPGAPANFSH 60
OAT2-tv2        MGFEELLEQVGGFGPFQLRNVALLALPRVLLPLHFLLPIFLAAVPAHRCALPGAPANFSH 60
OAT2-tv3        MGFEELLEQVGGFGPFQLRNVALLALPRVLLPLHFLLPIFLAAVPAHRCALPGAPANFSH 60
                ************************************************************

OAT2-tv1        QDVWLEAHLPREPDGTLSSCLRFAYPQALPNTTLGEERQSRGELEDEPATVPCSQGWEYD 120
OAT2-tv2        QDVWLEAHLPREPDGTLSSCLRFAYPQALPNTTLGEERQSRGELEDEPATVPCSQGWEYD 120
OAT2-tv3        QDVWLEAHLPREPDGTLSSCLRFAYPQALPNTTLGEERQSRGELEDEPATVPCSQGWEYD 120
                ************************************************************

OAT2-tv1        HSEFSSTIATE--WDLVCEQKGLNRAASTFFFAGVLVGAVAFGYLSDRFGRRRLLLVAYV 178
OAT2-tv2        HSEFSSTIATESQWDLVCEQKGLNRAASTFFFAGVLVGAVAFGYLSDRFGRRRLLLVAYV 180
OAT2-tv3        HSEFSSTIATE--WDLVCEQKGLNRAASTFFFAGVLVGAVAFGYLSDRFGRRRLLLVAYV 178
                ***********  ***********************************************

OAT2-tv1        STLVLGLASAASVSYVMFAITRTLTGSALAGFTIIVMPLELEWLDVEHRTVAGVLSSTFW 238
OAT2-tv2        STLVLGLASAASVSYVMFAITRTLTGSALAGFTIIVMPLELEWLDVEHRTVAGVLSSTFW 240
OAT2-tv3        STLVLGLASAASVSYVMFAITRTLTGSALAGFTIIVMPLELEWLDVEHRTVAGVLSSTFW 238
                ************************************************************

OAT2-tv1        TGGVMLLALVGYLIRDWRWLLLAVTLPCAPGILSLWWVPESARWLLTQGHVKEAHRYLLH 298
OAT2-tv2        TGGVMLLALVGYLIRDWRWLLLAVTLPCAPGILSLWWVPESARWLLTQGHVKEAHRYLLH 300
OAT2-tv3        TGGVMLLALVGYLIRDWRWLLLAVTLPCAPGILSLWWVPESARWLLTQGHVKEAHRYLLH 298
                ************************************************************

OAT2-tv1        CARLNGRPVCEDSFSQEAVSKVAAGERVVRRPSYLDLFRTPRLRHISLCCVVVWFGVNFS 358
OAT2-tv2        CARLNGRPVCEDSFSQEAVSKVAAGERVVRRPSYLDLFRTPRLRHISLCCVVVWFGVNFS 360
OAT2-tv3        CARPNGRPVCEDSFSQEAVSKVAAGERVVRRPSYLDLFRTPRLRHISLCCVVVWFGVNFS 358
                *** ********************************************************

OAT2-tv1        YYGLSLDVSGLGLNVYQTQLLFGAVELPSKLLVYLSVRYAGRRLTQAGTLLGTALAFGTR 418
OAT2-tv2        YYGLSLDVSGLGLNVYQTQLLFGAVELPSKLLVYLSVRYAGRRLTQAGTLLGTALAFGTR 420
OAT2-tv3        YYGLSLDVSGLGLNVYQTQLLFGAVELPSKLLVYLSVRYAGRRLTQAGTLLGAALAFGTR 418
                ****************************************************:*******

OAT2-tv1        LLVSSDMKSWSTVLAVMGKAFSEAAFTTAYLFTSELYPTVLRQTGMGLTALVGRLGGSLA 478
OAT2-tv2        LLVSSDMKSWSTVLAVMGKAFSEAAFTTAYLFTSELYPTVLRQTGMGLTALVGRLGGSLA 480
OAT2-tv3        LLVSSDMKSWSTVLAVMGKAFSEAAFTTAYLFTSELYPTVLRQTGMGLTALVGRLGGSLA 478
                ************************************************************

OAT2-tv1        PLAALLDGVWLSLPKLTYGGIALLAAGTALLLPETRQAQLPETIQDVERKSAPTSLQEEE 538
OAT2-tv2        PLAALLDGVWLSLPKLTYGGIALLAAGTALLLPETRQAQLPETIQDVERKSAPTSLQEEE 540
OAT2-tv3        PLAALLDGVWLSLPKLTYGGIALLAAGTALLLPETRQAQLPETIQDVERKRCVHRTVSVY 538
                ************************************************** .     .

OAT2-tv1        MPMKQVQN 546
OAT2-tv2        MPMKQVQN 548
OAT2-tv3        V------- 539
                :
```

Supplemental Figure 1. Amino acid alignment of OAT2-tv1, OAT2-tv2 and OAT2-tv3. The GenBank accession numbers for the peptide sequences corresponding to OAT2-tv1, OAT2-tv2 and OAT2-tv3 are NP006663, NP696961 and AAG43523, respectively. The amino acid alignment was performed with ClustalW2 (http://www.ebi.ac.uk/Tools/msa/clustalw2/).
