# Supplementary material for: Organic anion transporter 2 transcript variant 1 shows broad ligand selectivity when expressed in multiple cell lines
Source: Front Pharmacol. 2015 Oct 6;6:216. doi: 10.3389/fphar.2015.00216 (PMC4594013; doi:10.3389/fphar.2015.00216)
Supplement: Supplementary file 2 [file Image_2.PDF]

Supplemental Figure 2

```
OAT2-tv1   GIALLAAGTALLLPETRQAQLPETIQDVERKSAPTSLQEEEMPMKQVQN--- 546
OAT2-tv3   GIALLAAGTALLLPETRQAQLPETIQDVERKRCVHRTVSVYV---------- 539
mOAT2      GISFLAACTVLLLPETKKAQLPETIQDVERK----GRKIDRSGTELA----- 540
rOAT2      GIALVAACTALLLPETKKAQLPETIQDVERK----STQEEDV---------- 535
rbOAT2     GIALLAACTALLLPETRRAQLPETIQDVERK----SLQEEEMAMKQVQT--- 542
bOAT2      GIALLAACTALLLPETKQAQLPETIQDVERKSAPSSLQEEEMPMKQVQD--- 547
pOAT2      GIALLAACTALLLPETKQAQLPETIQDVERKSAPSSLQEEEMPMKQVQD--- 547
eqOAT2     GIALLAACTALLLPETRQAQLPETIQDVERKRLPSSLQEEEMSRKQVQD--- 546
opOAT2     GIALVASATALLLPETRHAELPETIQDVEKKRTLKGSQEEGTPMKVIRN--- 538
chkOAT2    GVAVFCGSVAFLLPETLNAHLPEGIQDIEKTQVKGPLQTSAPEGLQLQSLLK 540
```

Supplemental Figure 2. Amino acid alignment of the C-terminal end of OAT2 from human (OAT2-tv1 and OAT2-tv3), mouse (mOAT2), rat (rOAT2), rabbit (rbOAT2), cattle (bOAT2), pig (pOAT2), horse (eqOAT2), opossum (opOAT2) and chicken (chkOAT2). Amino acids in red are those that are conserved with the last 18 amino acids in OAT2-tv1 (shown in bold). The amino acid alignment was performed with ClustalW2 (http://www.ebi.ac.uk/Tools/msa/clustalw2/).
