# Supplementary material for: Organic anion transporter 2 transcript variant 1 shows broad ligand selectivity when expressed in multiple cell lines
Source: Front Pharmacol. 2015 Oct 6;6:216. doi: 10.3389/fphar.2015.00216 (PMC4594013; doi:10.3389/fphar.2015.00216)
Supplement: Supplementary file 3 [file Image_3.PDF]

Supplemental Figure 3

```
                                          TMH1
OAT2-tv1        MGFEELLEQVGGFGPFQLRNVALLALPRVLLPLHFLLPIFLAAVPAHRCALPGAPANFSH 60
OAT2-tv2        MGFEELLEQVGGFGPFQLRNVALLALPRVLLPLHFLLPIFLAAVPAHRCALPGAPANFSH 60
OAT1            MAFNDLLQQVGGVGRFQQIQVTLVVLPLLLMASHNTLQNFTAAIPTHHCRPP-ADANLS- 58
                *.*::**:****.* **  :*:*:.** :*:. *  *  * **:*:*:*  * * **:*

OAT2-tv1        QDVWLEAHLPREPDGTLSSCLRFAYPQALPNTTLGEERQSRGELEDEPATVPCSQGWEYD 120
OAT2-tv2        QDVWLEAHLPREPDGTLSSCLRFAYPQALPNTTLGEERQSRGELEDEPATVPCSQGWEYD 120
OAT1            KNGGLEVWLPRDRQGQPESCLRFTSPQWGLPFLNGTEANGTG------ATEPCTDGWIYD 112
                ::  **. ***: :*  .*****: **       * * :. *      ** **::** **

OAT2-tv1        HSEFSSTIATE--WDLVCEQKGLNRAASTFFFAGVLVGAVAFGYLSDRFGRR 170
OAT2-tv2        HSEFSSTIATESQWDLVCEQKGLNRAASTFFFAGVLVGAVAFGYLSDRFGRR 172
OAT1            NSTFPSTIVTE--WDLVCSHRALRQLAQSLYMVGVLLGAMVFGYLADRLGRR 162
                :* *.***.**  *****.::.*.: *.::::.***:**:.****:**:***
                                                  TMH2
```

Supplemental Figure 3. Amino acid alignment of the N-terminal end of OAT2-tv1, OAT2-tv2 and OAT1. The additional serine and glutamine in OAT2-tv2 are highlighted in red. The position of transmembrane helices 1 (TMH1) and 2 (TMH2) in a homology model of OAT1 structure (Perry et al., 2006) are denoted by the boxes. From this analysis, the additional two amino acids in OAT2-tv2 are expected to occur in the long extracellular loop. The amino acid alignment was performed with ClustalW2 (http://www.ebi.ac.uk/Tools/msa/clustalw2/).
